# Mucosal Metabolomic Profiling and Pathway Analysis Reveal the Metabolic Signature of Ulcerative Colitis

**DOI:** 10.3390/metabo9120291

**Published:** 2019-11-27

**Authors:** Joseph Diab, Terkel Hansen, Rasmus Goll, Hans Stenlund, Einar Jensen, Thomas Moritz, Jon Florholmen, Guro Forsdahl

**Affiliations:** 1 Natural Products and Medicinal Chemistry Research Group, Department of Pharmacy, Faculty of Health Sciences, University of Tromsø The Arctic University of Norway, 9037 Tromsø, Norway; Joseph.diab@uit.no (J.D.); Terkel.hansen@uit.no (T.H.); Einar.jensen@uit.no (E.J.); 2 Research Group of Gastroenterology and Nutrition, Department of Clinical Medicine, Faculty of Health Sciences, University of Tromsø The Arctic University of Norway, 9037 Tromsø, Norway; Rasmus.Goll@unn.no (R.G.); Jon.Florholmen@unn.no (J.F.); 3 Department of Medical Gastroenterology, University Hospital of North Norway, 9037 Tromsø, Norway; 4 Swedish Metabolomics Center, Department of Molecular Biology, Umeå University, 90736 Umeå, Sweden; hans.stenlund01@umu.se (H.S.); Thomas.Moritz@slu.se (T.M.); 5 The Novo Nordisk Foundation Center for Basic Metabolic Research, 2200 Copenhagen, Denmark

**Keywords:** inflammatory bowel disease, metabolomics, pathway analysis, ulcerative colitis, tryptophan metabolism, fatty acid metabolism, personalized treatment

## Abstract

The onset of ulcerative colitis (UC) is characterized by a dysregulated mucosal immune response triggered by several genetic and environmental factors in the context of host–microbe interaction. This complexity makes UC ideal for metabolomic studies to unravel the disease pathobiology and to improve the patient stratification strategies. This study aims to explore the mucosal metabolomic profile in UC patients, and to define the UC metabolic signature. Treatment- naïve UC patients (*n* = 18), UC patients in deep remission (*n* = 10), and healthy volunteers (*n* = 14) were recruited. Mucosa biopsies were collected during colonoscopies. Metabolomic analysis was performed by combined gas chromatography coupled to time-of-flight mass spectrometry (GC-TOF-MS) and ultra-high performance liquid chromatography coupled with mass spectrometry (UHPLC-MS). In total, 177 metabolites from 50 metabolic pathways were identified. The most prominent metabolome changes among the study groups were in lysophosphatidylcholine, acyl carnitine, and amino acid profiles. Several pathways were found perturbed according to the integrated pathway analysis. These pathways ranged from amino acid metabolism (such as tryptophan metabolism) to fatty acid metabolism, namely linoleic and butyrate. These metabolic changes during UC reflect the homeostatic disturbance in the gut, and highlight the importance of system biology approaches to identify key drivers of pathogenesis which prerequisite personalized medicine.

## 1. Introduction

Inflammatory bowel diseases (IBD) are chronic, relapsing inflammatory disorders in the gastrointestinal tract that affect around 0.3% of the population in Europe and North America with increasing worldwide incidence [1]. The two major forms of IBD, ulcerative colitis (UC) and Crohn’s disease (CD), are characterized by a dysregulated mucosal immune response triggered by several genetic and environmental factors in the context of host–microbe interaction [2]. The interaction between these components yield a network effect, defined as ‘IBD interactome’, which results in an overwhelming complexity [3]. This complexity cannot be solved by studying one component in isolation from the others. For instance, only 10% of IBD cases can be explained by genetic variance [4], while it remains unclear whether the alteration in the microbiota is primary or secondary to the chronic inflammation in IBD [5]. Recently, multiomics approaches were suggested as a tool to unravel the IBD interactome, and to improve the patient stratification strategies toward personalized medicine [3,6]. Metabolomics, defined as the comprehensive measurement of all metabolites (low-molecular-weight molecules) in a biological specimen, is perhaps the most closely linked to the phenotype [7]. It describes the pathophysiology of a disease at the molecular level, and provides predictive, prognostic and diagnostic markers of diverse disease states [8]. Previous published metabolomic data from IBD patients have given first hints on metabolic changes during the course of the disease. However, these data are either generated from serum [9,10,11,12,13,14], stool [12], or urine [15] samples. Moreover, these studies were restricted to pediatric patients [12,14], or treated patients [9,11,13,15].

This study aimed to explore the mucosal metabolomic profile in treatment-naïve UC patients compared to treated UC patients in deep remission and to healthy subjects. The high throughput metabolomic analysis was performed by a combined gas chromatography coupled to time-of-flight mass spectrometry (GC-TOF-MS) and ultra-high performance liquid chromatography coupled with mass spectrometry (UHPLC-MS) on biopsy samples. Our results maps the metabolic changes during IBD, and highlights the metabolic signatures of the IBD interactome network.

## 2. Results

### 2.1. Subjects Characteristics

Colon biopsies collected from newly diagnosed treatment-naïve UC patients (*n* = 18), UC patients in state of deep remission (*n* = 10), and healthy controls (*n* = 14) were included in this study. The study group characteristics are shown in Table 1. In addition, clinical data such as tumor necrosis factor (TNF) gene expression, levels of fecal calprotectin and C-reactive protein are provided. Furthermore, data on daily supplementation with omega-3 and previous treatment with antibiotics are included in Table 1.

### 2.2. Mucosal Metabolite Profiles in Treatment-Naïve UC Patients, UC Remission Patients and Controls

**Figure 1:**
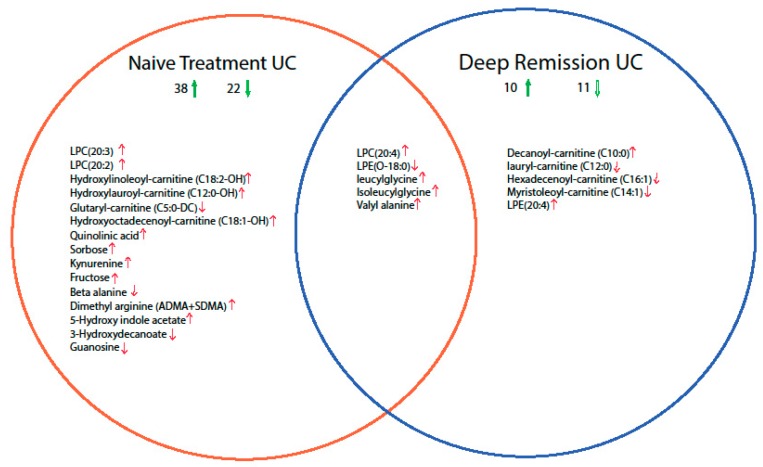
Venn diagram summarizing the comparison between the mucosal levels of metabolites in treatment-naïve ulcerative colitis (UC) patients and UC remission patients with healthy controls. Significantly altered metabolites were determined by Kruskal–Wallis test (False discovery rate (FDR) corrected *P* < 0.05), followed by the Dunn post-hoc test (Bonferroni adjusted *P* < 0.017). In total, the levels of 60 and 21 metabolites were changed in treatment-naïve UC and deep-remission UC, respectively, compared with healthy controls. The number of up/down regulated metabolites is indicated next to up/down green arrows. For simplicity, only the full names of significantly altered metabolites at a cut-off twofold change are presented. The red up/down arrows correspond to the direction of change (up/down regulation).

Mucosal metabolite profiles were compared to identify significant alteration in metabolite composition in treatment-naïve patients and UC deep remission patients compared with controls (Supplementary Table S1). The results are summarized as a Venn diagram in Figure 1. Among the 177 metabolites included in this study, the mucosal levels of 60 metabolites were altered in UC treatment-naïve patients compared with healthy controls. Among these metabolites, the mucosal levels of 38 metabolites were higher, and those of 22 metabolites were lower. Similarly, the mucosal levels of 21 metabolites were changed in UC remission patients compared with healthy controls. Accordingly, the mucosal levels of 10 metabolites were higher and those of 11 metabolites were lower. The most prominent changes among the study groups were in lysophospholipids, acyl carnitine, and amino acid profiles. In addition, 46 metabolites were changed in treatment-naïve UC patients compared to deep remission UC patients.

### 2.3. Discriminative Models for UC State

**Figure 2:**
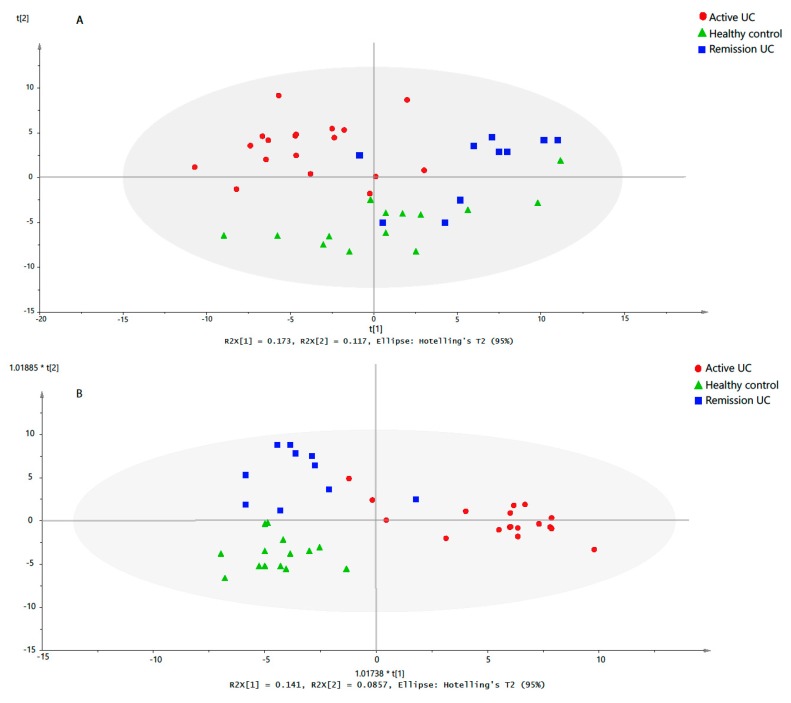
Multivariate analysis of the mucosal metabolomic profiles. Each subject was labeled according to the corresponding study group. (**A**) Principle component analysis (PCA) t1/t2-scores plots. The variation explained by PC1 and PC2 were 17.3% and 11.7%, respectively. t1 is the first component, which explains the largest variation, t2 is independent of t1 and explains second largest variation. (**B**) The t1/t2-score plot of the orthogonal partial least squares projection to latent structures-discriminant analysis (OPLS-DA) model (two predictive components and one orthogonal component) built from the mucosal metabolites profile of UC treatment-naïve patients, UC remission patients and healthy controls. t1 and t2 show the direction of class separation. The performance parameters *R*^2^X_cum_, *R*^2^Y_cum_ and Q^2^_cum_ were 0.33, 0.77 and 0.53, respectively.

Principle component analysis (PCA) was used as an unbiased multivariate analysis to have an overview of the variation within the data, to detect outliers, and to determine subgroups. The two main components explained 29% of the variability in the combined metabolomic data set (42 observations, 177 variables). Accordingly, the PCA t1/t2-scores plot (Figure 2A) revealed a distinct metabolomic profile in inflamed mucosa taken from treatment-naïve UC patients compared to noninflamed mucosa taken from UC remission patients and healthy controls. In addition, it was observed that the UC remission patients differed to a lesser extent from the healthy controls. Conversely, PCA did not show specific clustering patterns of the study subjects according to age, sex or activity score (Supplementary Figure S2).

**Figure 3:**
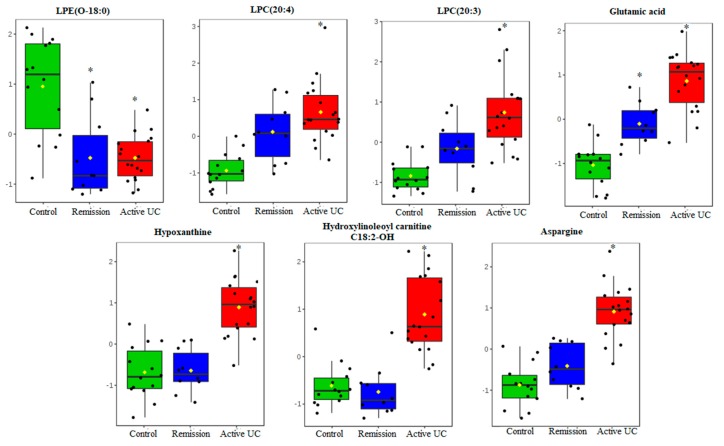
Jitter box plots of the mucosal level of the most discriminant metabolites (Variables importance of projection VIP score > 1.5 in OPLS-DA) between treatment-naïve UC, remission UC, and healthy controls. The levels of the metabolites were autoscaled for visualization. *P-value ≤ 0.017 versus healthy control was obtained by a Dunn post-hoc test.

A supervised orthogonal partial least squares projection to latent structures-discriminant analysis (OPLS-DA) model was built to identify the differential metabolites between active UC patients, remission UC patients and healthy controls. A significant OPLS-DA model (*P*-value from cross-validated analysis of variance (CV-ANOVA) was 6.15 × 10^−7^), with maximum separation between the study groups with good predictive ability Q^2^_cum_ >0.5 was obtained (Figure 2B). Additionally, a permutation test (Supplementary Figure S2) indicated that the obtained OPLS-DA model was not influenced by overfitting. The metabolites were ranked according to the variables importance in projection (VIP) scores (Supplementary Table S2) to identify the most distinctive metabolites between the study groups at a VIP threshold > 1.5. Glutamic acid, asparagine, lysophosphatidylethanolamine LPE (O-18:0), hypoxanthine, lysophosphatidylcholine LPC (20:3), hydroxyl carnitine, and LPC (20:4) were identified as the most important metabolites in the model, and the mucosal levels of these metabolites among the study groups are represented in Figure 3.

### 2.4. Pathway Analysis

**Figure 4:**
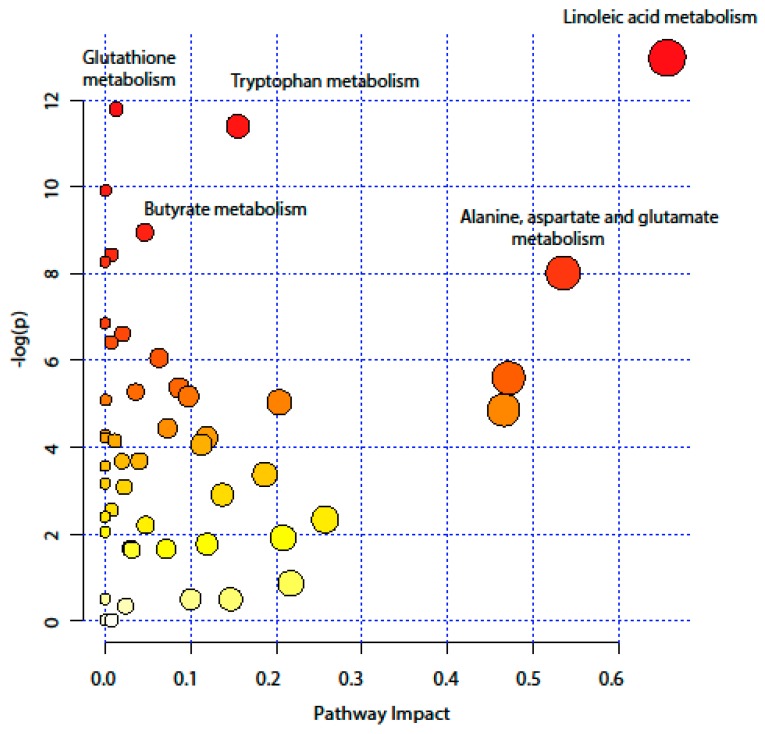
Pathway analysis, combining pathway enrichment and pathway topology analysis, of annotated metabolites in UC treatment-naïve patients and healthy controls. The x-axis marks the pathway impact and the y-axis represents the pathway enrichment. Each node marks a pathway, with larger sizes and darker colours represent higher pathway impact values and higher pathway enrichment.

Integrated pathway analysis was performed to capture the metabolic pathways disruption during the active UC state, and to ease the biological interpretation. The annotated metabolites were mapped into 50 metabolic pathways (Supplementary Table S3) according to the Kyoto encyclopedia of genes and genomes KEEG database. As Figure 4 shows, several pathways were the most perturbed, ranging from amino acid metabolism (such as tryptophan metabolism, and alanine, aspartate and glutamate metabolism) to antioxidant defense pathway (glutathione pathway). Furthermore, the pathway analysis revealed a disruption in the long- and short-chain fatty acid (LCFA and SCFA) metabolism, namely linoleic metabolism and butyrate metabolism. The impact value of altered metabolic pathways, based on topology analysis, ranged from 0.01–0.66. A summary of significantly altered pathways is provided in Table 2. In addition, the complete result from the pathway analysis containing all 50 metabolic pathways is provided in Supplementary Table S3.

## 3. Discussion

This study provides a unique and detailed snapshot of the mucosal metabolite profile in clearly stratified UC patients (treatment-naïve, newly diagnosed, and deep remission patients). The reported 177 metabolites revealed a distinctive metabolic fingerprint in active UC patients compared with healthy controls. In addition, the metabolomic profiling coupled with pathway analysis provided a deeper understanding of the metabolome changes among UC patients with ongoing active inflammation. Several metabolic pathways were identified, including pathways related to amino acid metabolism, SCFA and LCFA metabolism, and glutathione metabolism.

To our knowledge, this is the first study of mucosal metabolomic profile in treatment-naïve and deep remission UC patients. In contrast, previous studies were restricted to bio-fluids. It is well established that tissues are under greater homeostatic regulation than plasma^16^. Thus, it provides highly consistent measurements among individuals [16], and better understanding of the molecular basis of diseases [17]. Moreover, previous studies included treated and untreated UC patients. In the current work, however, only treatment- naïve UC patients were represented in the active UC group. In addition, the state of remission was defined by strict criteria (endoscopy, histology, and normalized TNF gene expression). Notably, remission patients were excluded from the pathway analysis. This stratification of patients allows capturing key metabolic alterations that are exclusively associated with the UC onset. Furthermore, the combination of two analytical metabolomic platforms allowed analysing metabolites in different polarity and molecular weight ranges, and gaining a wider prospective of the metabolome [18].

According to the pathway analysis, the omega-6 linoleic acid (ω-6 LA 18:2) metabolism had the highest impact score in the pathway analysis. Ω-6 LA, is an essential fatty acid, which is metabolised to dihomo-γ-linolenic acid (ω-6 DGLA 20:3). The latter is converted by fatty acid desaturase 1 (FADS1) to ω-6 arachidonic acid (AA 20:4) [19]. DGLA and AA are esterified with glycerol in the phospholipids, such as LPC, in the cell membrane, and released by phospholipase A2 during inflammation [19]. The released AA and DGLA are metabolised to form bioactive pro- and anti-inflammatory mediators. In the current data, LA was found to be lower in active UC patients. In contrast, the mucosal levels of LPC (20:3) and LPC (20:4) were higher in treatment-naïve UC compared to healthy controls, and were considered among the top discriminant metabolites between the study groups. This finding supports evidence suggesting that the onset of IBD is characterized by an imbalance between pro- and anti-inflammatory mediators [20]. For instance, the mucosal levels of AA related pro-inflammatory metabolites were elevated in treatment-naïve UC patients [21]. In addition, variations in the FADS1 gene were found to be associated with higher susceptibility to IBD [22,23]. Therefore, it seems that the increased metabolism of LA to AA is a crucial step in the IBD pathology.

Another important finding is the alteration in the amino acid metabolism, namely the tryptophan (Trp) metabolism and the alanine, aspartate and glutamate metabolism. Recently, Trp emerged as the hub of host–microbiota crosstalk considering that Trp metabolism pathways leading to serotonin, kynurenine (Kyn), and indole derivatives are under the direct or indirect control of the microbiota [24]. It was shown that supplementation with Trp improves the clinical symptoms and reduces the pro-inflammatory cytokines production in experimental colitis [25]. Furthermore, indole derivatives act as ligands for the aryl hydrocarbon receptor (AHR) inducing local production of interleukin-22 (IL-22), which maintains intestinal homeostasis, promotes immune defense and tissue repair. In the current study, we report a decreased mucosal level of Trp and an increased level of Kyn. This is in alignment with previous studies, which have reported low serum level of Trp in UC patients [10,12]. Notably, a large cohort study consisting of 148 UC patients has concluded that a higher Trp metabolism rate is associated with UC activity [26].

Furthermore, the current data demonstrates several perturbation in amino acid metabolism during UC. For instance, the mucosal levels of glutamic acid and asparagine were low in healed mucosa, and were gradually elevated in UC remission patients and active UC patients. Accordingly, glutamic acid and asparagine were discriminative between treatment-naïve UC patients, UC patients in remission and healthy controls. Interestingly, in a previous study, high levels of amino acids were detected in stool samples from IBD patients, and were linked with the gut microbiota dysbiosis [27]. In addition, higher urinary level of asparagine and glutamic acid were reported [28]. Notably, previous study of mucosal amino acids profile in IBD patients demonstrated increased levels of several amino acid, such as aspartate, glutamine, and glutamic acid in active UC patients [29]. However, we cannot determine to which degree the reported changes in mucosal amino acid levels are caused by gut microbiota.

Altered butyrate metabolism is another evidence of the bacterial dysbiosis in UC. It is well documented that the alteration in butyrate and other short chain fatty acid (SCFA) production is a hallmark of active UC patients [30]. For instance, it was found that dysbiosis in IBD patients is characterized by a decrease in the number of SCFAs/butyrate-producing bacteria [31]. Another study has reported reduction of butyrate and propionate in stool samples of IBD patients [32]. Although the current data did not show significant changes in butyrate related metabolites in the mucosa, the decreased mucosal level of glutamine in UC patients might indicate that glutamine is being used as energy source instead of butyrate, as previously reported [33]. Interestingly, previous data have shown low abundance of proteins related to this specific utilization of butyrate in UC patients’ mucosa [34].

The variation in the acylcarnitine profile, demonstrated in the current data, could also indicate energy impairment. Acylcarnitine is a mediator that transfers catabolism products of fatty acids and amino acids into mitochondria for β-oxidation [35]. This is a key step in the process of energy production. Therefore, the accumulation of medium and long chain fatty acyl carnitine, according to the current data, provides further evidence of the mitochondrial dysfunction. However, it is unclear yet whether the mitochondrial dysfunction in IBD is caused by a dysbiosis or if it is induced by the pro-inflammatory cytokines, such as TNF [36].

Although the inclusion criteria for remission patients was mucosal healing and immunological remission [37], the present work reveals a distinct metabolome in UC deep remission patients with respect to healthy controls and active UC patients. This comes in alignment with previously published data which reports a distinct mucosal lipid composition fingerprint in UC deep remission patients compared with healthy controls and treatment-naïve UC patients [38]. Consequently, from a clinical point of view, these findings supports the emerging importance of ‘Omics’ analysis in improving the current scoring system, monitoring the disease progression and improving the treatment strategies [39].

The relatively small sample size in the current study preclude subgroup analysis according to the severity of the disease. Hence, the reported results are exploratory and need to be validated by a larger cohort, which include inflamed and non-inflamed mucosa from UC patients. In addition, to further get insight in the mechanistic behind the alteration in the metabolic pathways, gene expression and/or protein data, preferably from the same patients, should be studied. Combining such multi omics data might also underline metabolite changes caused by the gut microbiota. Furthermore, we suggest the absolute quantification and identification of metabolites involved in the pathways of interest, especially tryptophan and butyrate pathways using targeted analysis. This is especially of interest for future evaluation of clinical validity, where absolute quantitative levels is a necessity. Suggestively, future studies also need to explore the relationship between metabolic changes, microbiota dysbiosis, and the activity of IBD. This approach will provide key insight into the disease outcome and response to treatment.

## 4. Materials and Methods

### 4.1. Patients and Biopsy Collection

Mucosal biopsies were collected from newly diagnosed treatment-naïve UC patients (*n* = 18) and UC patients in deep remission (*n* = 10). The UC diagnosis was made upon clinical, endoscopic and histological criteria established by the European Crohn and Colitis Organization (ECCO) guidelines [40]. The degree of inflammation was endoscopic evaluated by the scoring system of ulcerative colitis disease activity index (UCDAI); UCDAI score of 3–5 is defined as mild, 6–8 as moderate, and 9–12 as severe UC [41]. TNF-α mRNA expression levels were measured by real-time PCR in mucosal biopsies to evaluate the UC activity [42]. The state of deep remission was achieved after treatment with anti-TNF-α monoclonal antibody biologics. Deep remission was defined as endoscopic healed mucosa by ECCO 2017 consensus (Mayo score = 0) [43] and, additionally, normalized mucosal TNF-α level [44]. Subjects performing endoscopy for colonic cancer screening, with normal findings (no ulcer, no redness) and normal colonic histological examination, served as healthy controls (*n* = 14).

All biopsies were acquired from the rectum or sigmoid colon (Table 1). In active UC patients, biopsies were obtained from the most inflamed mucosa. The dry weight of the biopsies ranged from 2–8 mg. All biopsies were dry-frozen immediately at −80 °C, and kept at this temperature until further analysis. The Regional Committee of Medical Ethics of North Norway and the Norwegian Social Science Data Services approved the study and the storage of biological material under the number (REK NORD 2012/1349).

In addition, all enrolled subjects have signed an informed consent form, and the study was conducted in accordance with the Declaration of Helsinki.

### 4.2. Chemicals and Reagents

Detailed information of chemicals used for GC-MS and UHPLC-MS analysis is provided in the supplementary data section.

### 4.3. Sample Preparation

Metabolite extraction was carried out as previously described [45]. Briefly, each biopsy was transferred to an Eppendorf tube and kept on ice. Then, the extraction solution (methanol:water (8:1)) with all internal standards was added to the biopsy in a solid-to-solvent ratio of 1:15 (*w*/*v*). The final concentration of UPLC-MS standards and GC-MS standards was 0.625 ng/mL and 5 ng/μL respectively. Two tungsten beads were added to each tube, and the samples were shaken at 30 Hz for 3 min in a MM301 Vibration Mill (Retsch GmbH & Company KG). The beads were removed, and the samples were further centrifuged at 14,000 rpm and 4 °C for 3 min. Finally, the supernatant was transfer to a micro vials for UHPLC-MS and GC-MS analysis, 200 µL was used for UHPLC-MS analysis and 150 µL for GC-MS analysis. Samples were dried using a vacuum concentrator (MIVac, SP, Warminster, PA, USA). Quality Control (QC) samples were prepared by pooling 10 μL from each extract. Extracts were stored at −80 °C until analysis.

### 4.4. UHPLC-MS Analysis

On the day of analysis, samples were reconstituted in 20 µL of methanol:water (1:1) solution. The UHPLC-MS analysis was performed with an Infinity 1290 Agilent (Agilent Technologies, Santa Clara, CA, USA) ultra-high performance liquid chromatograph coupled with tandem mass spectrometry (UHPLC-MS-MS) as previously described [46]. Briefly, 1 µL of each extract was injected into the UHPLC system equipped with an Acquity column (HSS T3, 2.1 × 50 mm, 1.8 µm C18) in combination with a 2.1 mm × 5 mm, 1.7 µm VanGuard charged-surface hybrid (CSH) precolumn (Waters Corporation, Milford, MA, USA), held at 60 °C. Mobile phases used were MilliQ water with 0.1% formic acid (A) and 75:25 acetonitrile: 2-propanol with 0.1% formic acid (B). The following gradient was used: 10% B for 2 min, then B was increased to 99% in 5 min and held at 99% for 2 min. Subsequently, B was decreased to 0.1% in 0.3 min and the flow-rate was increased to 0.8 mL min^−1^ for 0.5 min. These conditions were held for 0.9 min, after which the flow-rate was reduced to 0.5 mL min^−1^ for 0.1 min before the next injection. Samples were randomly injected. The first parallel of extracts was analyzed in positive mode. Then, the instrument was switched to negative mode and the second parallel of extracts was injected. Blank samples with only methanol:water (1:1) solution were run prior and after each samples set. MS parameters were kept identical between the modes, with exception of the capillary voltage. The exact masses of metabolites were detected with an Agilent 6550 Q-TOF mass spectrometer equipped with an iFunnel jet stream electrospray ion source (Agilent Technologies, Santa Clara, CA, USA). The flow gas temperature was set at 150 °C, the drying gas flow at 16 L min^−1^ and the nebulizer pressure at 35 psi. The sheath gas temperature was set at 350 °C and the sheath gas flow was 11 L min^−1^. The capillary voltage was set at 4000 V for the positive mode and 4500 V for the negative mode. The *m*/*z* range was 70–1700, and data were collected in centroid mode with an acquisition rate of 4 scans/s. The QC-samples were a part of the quality control of the analysis were run in the beginning of the sample set. Auto MS/MS acquisition was used when running QC samples to generate MS/MS data.

### 4.5. GC-MS Analysis

Prior to injection, derivatization was performed as previously described [47]. Briefly, 30 µL of a methoxyamine solution in pyridine (15 μg μL^−1^) was added to the dry extract, and then shaken for 15 min on a shaking table. Derivatization was carried out at 70 °C for 1 h followed by room temperature for 16 h. Afterwards, the samples were trimethylsilylated (TMS) with 30 μL methyl-N-(trimethylsilyl) trifluoroacetamide MSTFA at room temperature for 1 h. Finally, 30 μL of heptane (including 15 ng methylstearate μL^−1^) was added and the vials were vortexed before 1 µL was injected splitless by a CTC Combi Pal autosampler (CTC Analytics AG, Switzerland) into an Agilent 6890 GC equipped with a fused silica capillary column (10 m × 0.18 mm I.D.) with a chemically bonded 0.18 µm DB 5-MS stationary phase (J&W Scientific, Folsom, CA, USA). Samples were randomly injected. Blank samples with only heptane were run prior and after the samples set. The injector temperature was 270 °C and the purge flow-rate was 20 mL min^−1^. The column temperature was set to 70 °C for 2 min, then increased to 320 °C by a rate of 40 °C min^−1^, and held there for 2 min using a gas flow rate of 1 mL min^−1^. The GC was coupled to the ion source of a Pegasus III TOF-MS (Leco Corp., St Joseph, MI, USA). The transfer line and MS instrument settings were as follows: Transfer lines and ion source temperature were set to 300 and 350 respectively. The mass detecting range was set to 50 to 800 *m*/*z*. An alkane series (C10-C40) was run together with all samples.

### 4.6. Metabolites Identification and Data Processing

Targeted feature extraction of the acquired UHPLC–MS data was performed using the Profinder™ software package, version B.08.00 (Agilent Technologies Inc., Santa Clara, CA, USA). In-house libraries with exact masses and experimental retention times were used for identification. The libraries contained metabolites from the following chemical classes: acylcarnitines, amino acids, carbohydrates, fatty acids, bile acids, nucleotides, small peptides, and lysophospholipids, namely lysophosphatidylcholine (LPC) and lysophosphatidylethanolamine (LPE). The allowed ion species for metabolites identification were ^+^H, ^+^Na, ^+^K, and ^+^NH_4_ in positive ionization mode, and –H, +HCOO in negative ionization mode. The mass tolerance was 10 ppm and the retention time tolerance 0.1 min. Only one charge for each metabolite was allowed. The extracted peaks were aligned and matched between samples, and then each compound was manually checked for mass and retention time agreement with the library. A two-step filtering approach was used for peak quality control: First, peaks with bad characteristics (e.g., overloaded, sample noise, non-Gaussian) were excluded from the analysis. Second, only peaks present in at least 75% of at least one study group were included.

Raw GC–MS data files were exported in NetCDF format to a MATLAB 8.3 (R2014a) (Mathworks, Natick, MA) based in-house script for baseline correction, chromatogram alignment, and peak deconvolution. Metabolite annotation was performed based on the retention index (RI) values and MS spectra from the in-house mass spectra library established by the Swedish Metabolomics Centre (Umeå, Sweden). The total number of annotated metabolites by UHPLC-MS and GC-MS was 128 and 66 respectively. Seventeen metabolites were detected with both methodologies, the signal detected with the UHPLC method was included in the statistical analyses. The UHPLC–MS metabolites were normalized by the total peak areas, whereas GC–MS metabolites were normalized by internal standards as described before [48]. A combined data set containing 177 metabolites was submitted to statistical analysis.

### 4.7. Statistical Analysis

Statistical analysis was carried out using RStudio: Integrated Development Environment (version 1.0.143). Undetectable Metabolites, which represented 0.6% of the total reported metabolites, were assigned a value corresponding to half of the minimum positive value in the original data. Shapiro–Wilk test of normality was applied, and the data was not found normally distributed. Kruskal–Wallis one way analysis of variance test was performed to compare the mean concentration of metabolites between treatment-naïve UC, remission UC, and control groups. Acquired *p-*values were adjusted using Benjamini and Hochberg FDR method [49]. Dunn’s test [50] was applied as a post-hoc test, and significant *p*-value cut-off was corrected to 0.017 by Bonferroni multiple comparison method [51]. Multivariate analysis was carried out using SIMCA software (version 14.0.0.135559; Sartorius AB, Umea, Sweden). The metabolites were auto-scaled and mean-centered in order to adjust the importance of high and low abundance metabolites to an equal level [52]. Unsupervised PCA was first performed to assess the unicity of the metabolome for each of the study groups. Then, supervised OPLS-DA [53] was employed and metabolites were classified according to corresponding regression coefficients to identify the most important metabolites in discriminating between the study groups. The parameters of the OPLS-DA model were described by *R*^2^X_cum_, *R*^2^Y_cum_ and Q^2^_cum_, whereas, *R*^2^X_cum_ is the cumulative modeled variation in X, *R*^2^Y_cum_ is the amount of variation in X correlated to Y (response matrix) and Q^2^_cum_ is the cumulative predicted ability of the model [54]. The validity and degree of overfitting of the OPLS-DA model was assessed by conducting analysis of variance testing of cross-validated predictive residuals (CV-ANOVA), and permutation analyses.

Pathway analysis was performed using MetaboAnalyst 4.0, a web tool for metabolomics data analysis (http://www.metaboanalyst.ca/) [55]. First, all 177 metabolites were annotated according to ‘Human Metabolome Database’ (HMDB) [56] and linked to a metabolic pathway according to KEGG database [57]. Secondly, powerful pathway enrichment analysis coupled with pathway topology analysis was carried out to identify the altered metabolic pathways in active UC compared with healthy state. The enrichment analysis was based on a global test [58] while, the node/metabolite importance was measured by relative betweenness centrality [59]. Obtained *P-*values from the enrichment analysis were adjusted by Holm method [60]. Adjusted *P-*values lower than 0.05 were considered significant.

## 5. Conclusions

The present report provides an in-depth description of the mucosal metabolome in UC via a high-throughput metabolomic analysis of colon biopsies taken from UC treatment-naïve patients, UC patients in state of deep remission, and healthy subjects. The study of mucosal metabolites revealed the main metabolic signatures in active UC, and reflects the homeostatic disturbance in the gut. The reported metabolites were identified by searching the human-only metabolites database, and only human metabolic pathways were included in the pathway analysis. However, the gut microbiota seems to be heavily involved in altering several metabolic pathways in the colon mucosa. This highlights the importance of integrating IBD-ome compartments by system biology approaches to identify key drivers of pathogenesis that require personalized treatment.

## Figures and Tables

**Table 1:** Description of study group characteristics.

Characteristics	Active UC (Debut)	UC Remission	Healthy Controls
Number of Subjects	18	10	14
Age, years (mean, range, *P*-value *)	40 (20–68) 0.09	48 (31–77) 0.18	55 (26–83)
Gender (Female/Male)	6/12	4/6	4/10
UCDAI Score (Mild, Moderate, Severe)	12/2/4		
Biopsy sampling site (Rectum/sigmoid)	3/15	5/5	4/10
TNF-α, copies/μg of total RNA (mean, range, *P*-value *)	18,122 (4600–31,700) 0.01	4675 (800–7300) 0.11	5478 (1800–11,300)
Fecal calprotectin, μg/g (mean, range, *P*-value *)	828 (25–1970) < 0.01	53 (25–150) 0.15	46 (25–180)
C-Reactive protein, mg/L (mean, range, *P*-value *)	16.5 (5–92) 0.08	5.6 (5–11) 0.31	5.2 (5–11)
Smoking/non-smoking	1/17	1/9	3/11
Omega-3 daily supplementation (Yes/No)	6/12	3/7	7/7
Antibiotic in the last 6 months prior to the biopsy (Yes/No)	3/15	0/10	2/12

* computed *P*-value from the comparison of two means versus healthy controls group.

**Table 2:** Altered metabolic pathways according to pathway analysis.

KEEG Pathway	Numb. Metabolites	Matched Metabolites from the Metabolomics Data	Adjusted *P*-value **	Impact ***
Linoleic Acid Metabolism	15	Linoleic acid *	<0.001	0.66
Alanine, Aspartate and Glutamate Metabolism	24	N-Acetyl-L-aspartic acid *; L-Asparagine *; L-Glutamine *; L-Glutamic acid *; Gamma-Aminobutyric acid; Fumaric acid; Succinic acid	0.014	0.53
Tryptophan Metabolism	79	L-Tryptophan *; 5-Hydroxyindoleacetic acid *; L-Kynurenine *; Picolinic acid; Quinolinic acid*	<0.001	0.15
Butyrate Metabolism	40	Gamma-Aminobutyric acid; L-Glutamic acid *; Fumaric acid	0.006	0.05
Glutathione Metabolism	38	L-Glutamic acid *; Cysteinylglycine; Pyroglutamic acid *; Ornithine *	<0.001	0.01

* Altered metabolites (*P*-value ≤ 0.017 versus healthy control obtained by Dunn post-hoc test). ** *P*-values were calculated from the enrichment analysis then adjusted by Holm method. *** Impact is the pathway impact score calculated from pathway topology analysis.

## Supplementary Materials

The following are available online at https://www.mdpi.com/2218-1989/9/12/291/s1, Figure S1: Multivariate analysis of the mucosal metabolomic profiles according to the subjects’ sex, age, and activity score., Figure S2: Permutation test of the OPLS-DA model, Table S1: Kruskal Wallis analysis comparing the mucosal metabolomic profile among the study groups, Table S2: The variables importance in projection (VIP) scores of the 177 metabolites included in this study according to the OPLS-DA model, Table S3: Pathway analysis of the 177 metabolites included in this study in active UC compared with healthy controls.
