# Supplementary material for: Mucosal Metabolomic Profiling and Pathway Analysis Reveal the Metabolic Signature of Ulcerative Colitis
Source: Metabolites. 2019 Nov 27;9(12):291. doi: 10.3390/metabo9120291 (PMC6950742; doi:10.3390/metabo9120291)
Supplement: Supplementary file 1 [file metabolites-09-00291-s001.zip › supplementary materials/Sup Table 1.pdf]

Kruskal Wallis analysis comparing the mucosal metabolomic profile among the study groups

Metabolites	Kruskal Wallis Test adj. p-value*	Active UC vs Healthy Control		Active UC vs Remission UC		Remission UC vs Healthy Control	
		Fold change	P.value**	Fold change	P.value**	Fold change	P.value**
2-Hydroxyhexanoate	1.000	0.90	0.804	0.82	0.218	0.91	0.166
2-Hydroxyoctanoate	0.297	0.75	0.259	1.18	0.217	1.58	0.032
2-Hydroxypalmitate	0.503	0.92	0.360	1.11	0.247	1.20	0.059
2-Hydroxystearate	**0.028**	1.15	**0.003**	1.31	0.622	1.14	0.037
2'-O-methylguanosine	0.153	1.95	0.769	1.31	0.035	0.67	0.024
3-Carboxy-4-methyl-5-propyl-2-furanpropanoate (CMPF)	**0.014**	0.31	0.529	0.50	**0.001**	1.60	**0.012**
3-Hydroxydecanoate	**0.021**	0.70	**0.015**	0.43	**0.005**	0.62	0.562
3-Hydroxylaurate	0.221	0.72	0.216	1.29	0.202	1.80	0.023
3-Hydroxymyristate	0.775	0.82	0.377	1.15	0.355	1.41	0.100
3-Hydroxypalmitate	0.291	0.87	0.296	1.12	0.186	1.28	0.031
3-methylglutarylcarnitine	**0.003**	0.61	**0.001**	0.59	**0.005**	0.97	0.818
3-isomethylglutarylcarnitine	**0.003**	0.60	**0.001**	0.59	**0.004**	0.99	0.897
4-Aminobutyric Acid	1.000	0.82	0.305	1.46	0.703	1.78	0.213
5-hydroxyhexanoate	1.000	0.90	0.765	0.78	0.270	0.86	0.191
5-hydroxyindoleacetate	**<0.001**	2.44	**<0.001**	2.81	**0.002**	1.15	0.520
5-methylthioadenosine	1.000	0.73	0.495	0.80	0.357	1.09	0.772
5-oxoproline	**<0.001**	1.69	**<0.001**	1.99	**0.002**	1.18	0.520
Acetylcarnitine (C2:0)	**0.021**	0.73	0.151	1.14	0.045	1.55	**0.002**
acisoga	1.000	0.85	0.833	1.03	0.646	1.22	0.536
Aconitic Acid	1.000	0.50	0.314	0.54	0.453	1.09	0.879

Allothreonine	0.342	1.01	0.061	1.23	0.920	1.22	0.088
Arabinose	1.000	1.08	0.486	0.85	0.783	0.79	0.736
Arachidonate (20:4)	**0.033**	1.18	**0.007**	1.21	0.021	1.02	0.915
Arginine	1.000	1.08	0.279	1.02	0.535	0.95	0.734
Aspargene	**<0.001**	1.48	**<0.001**	1.78	**0.001**	1.20	0.215
Aspartylleucine	**0.001**	2.20	**0.007**	1.52	**<0.001**	0.69	0.172
Beta-alanine	**<0.001**	0.64	**<0.001**	0.43	**0.004**	0.68	0.426
Butyrylcarnitine (C4:0)	0.318	1.14	0.136	1.23	0.049	1.08	0.551
Carnitine	**0.032**	0.57	0.018	0.68	**0.008**	1.18	0.631
Cellobiose	1.000	1.41	0.397	0.73	0.691	0.52	0.268
Cervonyl-carnitine (C22:6)	0.624	1.35	0.650	0.84	0.163	0.62	0.086
Citric Acid	0.762	0.75	0.098	1.13	0.513	1.50	0.423
Citrulline	**<0.001**	1.68	**<0.001**	1.56	**0.001**	0.93	1.000
Creatine	**0.026**	0.85	**0.010**	0.82	**0.010**	0.97	0.826
Cysteine-glycine	1.000	0.87	0.532	1.11	0.402	1.28	0.182
Cysteine-glutathione disulfide	0.148	0.90	0.053	1.57	0.537	1.74	0.024
Decanoylcarnitine (C10:0)	**0.017**	0.68	0.026	2.14	0.218	3.17	**0.002**
Dimethylarginine (ADMA + SDMA)	**<0.001**	1.85	**<0.001**	2.09	**0.002**	1.13	0.696
Docosahexaenoate (DHA; 22:6)	0.437	1.14	0.253	0.91	0.311	0.80	0.051
Docosapentaenoate (DPA; 22:5)	0.142	1.25	0.026	1.28	0.063	1.03	0.882
Docosapentaenoate (n6 DPA; 22:5)	**0.002**	1.88	**0.004**	1.54	**0.001**	0.82	0.475
Dodecanoic Acid	1.000	0.81	0.948	0.58	0.432	0.72	0.489
Eicosadieneoyl-carnitine (C20:2)	0.122	1.68	0.409	1.08	0.012	0.65	0.090
Eicosanoic Acid	1.000	1.05	0.423	0.80	0.563	0.76	0.215
Eicosapentaenoate (EPA; 20:5)	**0.018**	0.79	**0.001**	0.52	0.226	0.65	0.110
Eicoseneoyl-carnitine (C20:1)	0.169	1.59	0.506	0.82	0.064	0.51	0.019
Fructose	**0.023**	0.63	**0.002**	2.21	0.141	3.52	0.204
Fumaric Acid	0.226	1.09	0.036	1.23	0.101	1.13	0.811

Galactose	0.076	0.49	0.086	0.61	0.009	1.23	0.315
Gamma-glutamylisoleucine	**0.001**	2.48	**0.016**	1.50	**<0.001**	0.60	0.098
Gamma-glutamylmethionine	**0.004**	2.10	**0.007**	1.67	**0.001**	0.80	0.368
Gamma-glutamylphenylalanine	**0.001**	2.29	**0.008**	1.54	**<0.001**	0.67	0.203
Gamma-glutamylvaline	**0.001**	2.16	**0.010**	1.50	**<0.001**	0.69	0.159
Glucose	**0.038**	0.56	0.069	0.68	**0.004**	1.21	0.245
Glutamic Acid	**<0.001**	1.33	**<0.001**	1.94	0.017	1.46	0.017
Glutarylcarnitine (C5:0-DC)	**0.016**	0.45	**0.006**	0.27	**0.008**	0.60	0.890
Glycerol-3-Phosphate	**0.019**	0.70	**0.002**	0.54	0.165	0.76	0.160
Guanidinosuccinate	0.067	0.70	0.228	1.07	0.072	1.53	0.006
Guanosine	**<0.001**	0.27	**0.002**	0.36	0.000	1.34	0.141
Heptadecanoic Acid	1.000	1.09	0.505	1.11	0.612	1.02	0.928
Hexadecadienoyl-carnitine (C16:2)	0.263	1.47	0.763	1.02	0.033	0.69	0.077
Hexadecanoic Acid	1.000	0.91	0.250	0.77	0.669	0.84	0.560
Hexadecenoyl-carnitine (C16:1)	**0.016**	2.06	0.778	1.03	**0.002**	0.50	**0.007**
Hexanoylcarnitine (C6:0)	0.313	2.03	0.208	1.47	0.038	0.72	0.374
Homoarginine	**0.045**	1.20	**<0.001**	1.16	0.017	0.97	0.860
Hydroxybutyrylcarnitine (C4:0-OH)	0.260	0.34	0.623	0.92	0.030	2.70	0.099
Hydroxyisovaleroylcarnitine (C5:0-OH)	1.000	1.40	0.401	1.27	0.149	0.91	0.514
Hydroxylauroyl-carnitine (C12:0-OH)	**<0.001**	2.46	**<0.001**	3.17	**0.002**	1.29	0.527
Hydroxylinoleoyl-carnitine (C18:2-OH)	**<0.001**	2.90	**<0.001**	2.52	**<0.001**	0.87	0.532
Hydroxymyristate	0.264	0.75	0.444	1.11	0.110	1.48	0.029
Hydroxymyristoyl-carnitine (C14:0-OH)	**<0.001**	2.39	**0.003**	1.76	**<0.001**	0.74	0.117
Hydroxyoctadecenoyl-carnitine (C18:1-OH))	**<0.001**	3.20	**0.002**	2.08	**<0.001**	0.65	0.169
Hydroxypalmitoleoyl-carnitine (C16:1-OH)	**<0.001**	2.20	**<0.001**	1.83	**<0.001**	0.83	0.459

Hydroxystearate	**0.030**	1.17	**0.003**	1.34	0.607	1.15	0.041
Hypoxanthin	**<0.001**	1.14	**0.001**	1.10	**<0.001**	0.97	0.242
Inosine	1.000	0.76	0.532	1.17	0.402	1.54	0.182
Inositol	0.089	0.82	0.055	0.85	0.015	1.04	0.501
Isobutyrylcarnitine (C4:0)	0.311	1.15	0.140	1.22	0.046	1.07	0.529
Isocitric Acid	0.613	0.71	0.077	1.19	0.610	1.68	0.299
Isoleucylglycine	**0.032**	0.92	**0.007**	2.32	0.836	2.51	0.012
Isovalerylcarnitine (C5:0)	0.160	2.02	0.270	1.26	0.016	0.63	0.176
Kynurenine	**<0.001**	2.46	**<0.001**	2.97	**0.003**	1.20	0.453
L-glutamine	**0.002**	0.64	0.371	1.10	**0.002**	1.73	**<0.001**
L-isoleucine	**0.035**	1.08	0.176	0.96	0.057	0.88	**0.003**
L-valine	**<0.001**	1.48	**<0.001**	1.68	**0.001**	1.13	0.786
Laminaribiose	0.977	1.30	0.219	0.65	0.764	0.50	0.179
Laurate (12:0)	0.091	0.61	0.770	1.06	0.022	1.72	0.015
Laurylcarnitine (C12:0)	**0.006**	7.51	0.333	1.51	**<0.001**	0.20	**0.012**
leucine	**0.039**	1.08	0.181	0.96	0.059	0.88	**0.003**
leucylglycine	**0.032**	0.84	**0.014**	2.30	0.523	2.75	**0.006**
Linoleate (18:2)	**0.001**	0.77	**<0.001**	0.60	0.063	0.78	0.080
Linolenate (18:3)	0.442	0.75	0.225	0.79	0.059	1.05	0.449
Linoleneoyl-carnitine (C18:3)	0.230	1.61	0.321	1.17	0.024	0.73	0.193
Linoleoylcarnitine (C18:2)	0.171	1.63	0.590	0.89	0.018	0.54	0.075
LPC(14:0)	**0.043**	0.89	0.028	1.28	0.370	1.43	**0.006**
LPC(15:0)	0.190	0.85	0.645	1.06	0.053	1.24	0.025
LPC(16:0)	**0.005**	0.91	0.211	0.96	**<0.001**	1.05	0.020
LPC(16:1)	0.157	1.19	0.025	1.18	0.080	0.99	0.798
LPC(17:0)	**0.002**	0.83	**<0.001**	0.71	0.090	0.85	0.085
LPC(18:0)	0.409	0.93	0.229	1.06	0.322	1.13	0.048
LPC(18:1)	**0.015**	1.30	0.615	0.94	**0.006**	0.73	**0.002**

LPC(18:3n3)	1.000	0.90	0.566	1.01	0.442	1.12	0.811
LPC(19:0)	**0.044**	0.79	0.160	1.21	0.076	1.54	**0.004**
LPC(20:0)	0.183	0.64	0.250	0.71	0.019	1.10	0.211
LPC(20:1n9)	**0.007**	1.27	0.009	1.24	**0.002**	0.98	0.451
LPC(20:2)	**<0.001**	2.10	**<0.001**	2.03	**<0.001**	0.97	0.917
LPC(20:3)	**<0.001**	1.42	**<0.001**	2.10	0.031	1.47	0.040
LPC(20:4)	**<0.001**	1.26	**<0.001**	2.51	0.237	2.00	**0.003**
LPC(20:5n3)	1.000	0.80	0.684	0.90	0.304	1.13	0.529
LPC(22:5n6)	**0.001**	1.57	**<0.001**	1.76	0.020	1.12	0.258
LPC(22:6)	0.674	1.11	0.086	1.00	0.385	0.91	0.516
LPE(16:0)	**0.006**	0.98	**0.001**	0.83	0.968	0.84	**0.005**
LPE(18:0)	0.343	1.27	0.802	1.01	0.046	0.80	0.092
LPE(18:2)	0.360	0.81	0.143	0.84	0.056	1.04	0.578
LPE(20:3)	**0.004**	1.04	**<0.001**	1.82	0.602	1.76	**0.011**
LPE(20:4)	**0.001**	0.90	**0.001**	1.95	0.575	2.17	**<0.001**
LPE(20:5n3)	1.000	0.66	0.632	0.74	0.202	1.13	0.423
LPE(O-16:0)	**0.006**	1.03	**0.001**	0.76	0.945	0.74	**0.006**
LPE(O-18:0)	**0.001**	1.00	**<0.001**	0.50	0.705	0.50	**0.001**
Lysine	0.903	0.97	0.164	1.00	0.941	1.03	0.205
Lyxose	1.000	1.16	0.200	1.04	0.651	0.89	0.501
Malic Acid	0.652	1.04	0.081	1.20	0.525	1.15	0.370
Maltose	1.000	1.41	0.339	0.73	0.806	0.52	0.290
Mead Acid (20:3)	**0.002**	1.34	0.001	1.22	**0.003**	0.91	0.985
Methionine	1.000	1.03	0.916	0.98	0.818	0.95	0.757
Methylmalonyl carnitine	**0.007**	0.43	0.027	0.56	**0.001**	1.28	0.211
Myristic Acid	1.000	0.84	0.297	0.80	0.368	0.95	0.969
Myristoleoylcarnitine (C14:1)	**0.028**	2.36	0.726	1.07	**0.003**	0.45	**0.013**
Myristoylcarnitine (C14:0)	**0.034**	2.04	0.452	1.11	**0.003**	0.54	0.030

N-Acetyl-Glucosamine	0.132	0.83	0.083	0.86	0.019	1.04	0.461
N- Acetyl -L-Aapartic Acid	**0.007**	1.34	**0.001**	1.68	0.081	1.26	0.189
N6-succinyladenosine	0.944	0.81	0.403	0.78	0.134	0.96	0.480
Nicotinamide	**0.001**	1.64	**<0.001**	1.84	**0.007**	1.12	0.449
Nonanoic Acid	1.000	1.00	0.949	0.36	0.254	0.36	0.254
Octadecadienoic Acid	**0.009**	1.09	0.017	0.83	0.200	0.76	**0.001**
Octadecanedioate (C18)	0.133	0.67	0.777	0.86	0.017	1.28	0.042
Octadecenoic Acid	1.000	1.10	0.375	0.45	0.586	0.41	0.200
Octanoyl-carnitine (C8:0)	0.097	2.26	0.019	2.39	0.047	1.06	0.895
Octenoyl-carnitine (C8:1)	**0.011**	0.54	0.212	0.83	**0.001**	1.53	0.035
Oleate (18:1)	**0.017**	0.82	**0.002**	0.73	0.055	0.89	0.383
Oleoylcarnitine (C18:1)	0.069	1.66	0.938	0.95	0.011	0.57	0.018
Ophthalmic Acid	**0.004**	0.53	**0.005**	0.60	**0.001**	1.14	0.503
Ornithine	**0.019**	1.47	**0.003**	1.63	0.033	1.10	0.583
Palmitate (16:0)	1.000	0.87	0.529	0.95	0.142	1.09	0.391
Palmitoleate (16:1)	0.781	0.72	0.818	1.18	0.161	1.63	0.125
Palmitoylcarnitine (C16:0)	0.051	1.31	0.101	0.80	0.135	0.61	0.005
LPC(14:0)	0.075	0.88	0.059	1.19	0.297	1.36	0.009
Pentadecanoic Acid	1.000	0.76	0.872	0.92	0.181	1.21	0.256
Phenylacetylglutamine	1.000	1.11	0.490	1.38	0.626	1.25	0.290
Phenylalanine	0.308	1.17	0.091	1.14	0.063	0.97	0.751
Phenylalanylglutamate	**0.001**	2.28	**0.008**	1.53	**<0.001**	0.67	0.167
Phenylalanylleucine	0.933	0.76	0.181	0.80	0.881	1.05	0.196
Phenylalanylphenylalanine	0.697	0.83	0.139	1.14	0.839	1.38	0.143
Picolinic Acid	1.000	1.00	0.711	1.18	0.858	1.18	0.625
Proline	0.763	1.00	0.131	1.14	0.978	1.14	0.185
Propionylcarnitine (C3:0)	0.102	1.43	0.988	0.90	0.017	0.63	0.023
Pyroglutamic Acid	**0.003**	1.07	**<0.001**	1.36	0.169	1.27	0.055

Quinolinic Acid	0.059	2.84	0.007	5.00	0.069	1.76	0.566
Ribose	**0.005**	1.43	**0.001**	1.53	**0.013**	1.07	0.581
S-adenosylhomocysteine (SAH)	**0.003**	0.78	**0.007**	1.50	0.175	1.91	**<0.001**
Sakebiose	1.000	1.41	0.413	0.73	0.683	0.52	0.274
Sorbose	**0.005**	0.52	**<0.001**	2.46	0.137	4.77	0.100
Squalene	1.000	0.76	0.439	1.02	0.689	1.35	0.295
Stearoylcarnitine (C18)	0.574	1.17	0.240	0.75	0.418	0.64	0.075
Suberate (octanedioate)	0.393	0.71	0.856	0.97	0.076	1.37	0.065
Succinic Acid	0.196	0.89	0.019	0.71	0.342	0.80	0.269
Sucrose	1.000	0.57	0.248	1.25	0.762	2.19	0.200
Tetradecadienyl-carnitine (C14:2)	0.437	1.74	0.819	1.14	0.060	0.66	0.111
Threonine	0.324	1.02	0.048	1.24	0.918	1.22	0.109
Tiglyl carnitine (C5:1)	0.079	1.84	0.235	1.21	0.007	0.66	0.123
Tryptophan	**0.002**	0.67	**0.001**	0.68	**0.003**	1.01	0.975
Tryptophan betaine	0.767	0.74	0.478	1.66	0.276	2.23	0.099
Tyrosine	0.220	1.25	0.027	1.33	0.164	1.06	0.560
Uracil	**<0.001**	0.48	**0.002**	0.55	**<0.001**	1.14	0.285
Valerylcarnitine (C5:0)	0.311	2.32	0.538	1.47	0.034	0.63	0.138
valylalanine	**0.022**	0.84	**0.009**	2.32	0.578	2.75	**0.005**
Xylose	1.000	1.13	0.163	0.95	0.582	0.84	0.500

* Kruskall Wallis p values adjusted by Benjamini-Hocheberg method
** Dunn post hoc test p values
