# Supplementary material for: Mucosal Metabolomic Profiling and Pathway Analysis Reveal the Metabolic Signature of Ulcerative Colitis
Source: Metabolites. 2019 Nov 27;9(12):291. doi: 10.3390/metabo9120291 (PMC6950742; doi:10.3390/metabo9120291)
Supplement: Supplementary file 1 [file metabolites-09-00291-s001.zip › supplementary materials/Supp data.pdf]

Supplementary Material

1- Chemicals

All Solvents and chemicals were of HPLC grade or higher. The isotope labelled Internal standards (IS) for LC-MC, phenylalanine ($^{13}C_9$), caffeine (trimethyl-$^{13}C_3$), cholic acid (2,2,4,4-d_4) were obtained from Cambridge Isotope Laboratories, arachidonic acid-d_8 was purchased from Ladoran (Karolinska Institute, Stockholm, Sweden) and caffeic Acid-$^{13}C_9$ was aquired from Sigma-Aldrich (St. Louis, MO, USA). IS for GC-MS, proline-($^{13}C_5$), succinic acid-($^2H_4$), glutamic acid-($^{13}C_5$,^{15}N), myristic acid-(1,2,3-$^{13}C_3$), cholesterol-($^2H_7$) and disodium α- ketoglutarate-($^{13}C_4$) were ordered from Cambridge Isotope Laboratories (Andover, MA). Sucrose-($^{13}C_{12}$), palmitic acid-($^{13}C_4$) and butanediamine·2HCl -($^2H_4$) were from Campro (Veenendaal, The Netherlands). Glucose-($^{13}C_6$) was from Aldrich (Steinheim, Germany) and salicylic acid-($^2H_6$) was from Icon (Summit, NJ, USA). Methyl stearate was purchased from Sigma (St. Louis, USA). N-Methyl-N-(trimethylsilyl) trifluoroacetamide (MSTFA), 1% trimethylchlorosilane (TMCS) and pyridine (silylation grade) were acquired from ThermoScientific (Rockford, IL, USA), heptane was purchased from Fischer Scientific (Loughborough, UK). Stock solutions of the IS were prepared in either Milli-Q water or methanol from J.T. Baker (Deventer, Holland) at the same concentration, 0.5 μg μL-1. Water was purified using a MilliQ gradient system (Millipore, Milford, MA, USA). Acetonitrile (ACN) was obtained from Merck (Darmstadt, Germany). Isopropanol was from VWR PROLABO (Fontenay-sous-Bois, France).

2. Supplementary Figures

Supplementary Figure 1. Three PCA t1/t2-scores plots for mucosal metabolite profiles. The variation explained by PC1 and PC2 were 17.3% and 11.7%, respectively. t1 is the first component, which explains the largest variation, t2 is independent of t1 and explains second largest variation which is orthogonal to t1. The study subjects in Supplementary figure 1.A, 1.B, and 1C were colored according to sex, UC disease activity, and age respectively.

Supplementary Figure 2. OPLS-DA permutation plot for the metabolomic data set displaying the correlation coefficients between the original Y variable (naïve treatment UC, remission UC, and healthy controls) and the permuted Y variable on the x-axis versus the cumulative R^2Y and Q^2 on the y-axis, with the regression line between them. The intercept is the measure of the over fit. The Y-axis intercept below 0.5 for R^2Y and below 0.05 for Q^2.
