# Supplementary figures and images for: Mucosal Metabolomic Profiling and Pathway Analysis Reveal the Metabolic Signature of Ulcerative Colitis
Source: Metabolites. 2019 Nov 27;9(12):291. doi: 10.3390/metabo9120291 (PMC6950742; doi:10.3390/metabo9120291)

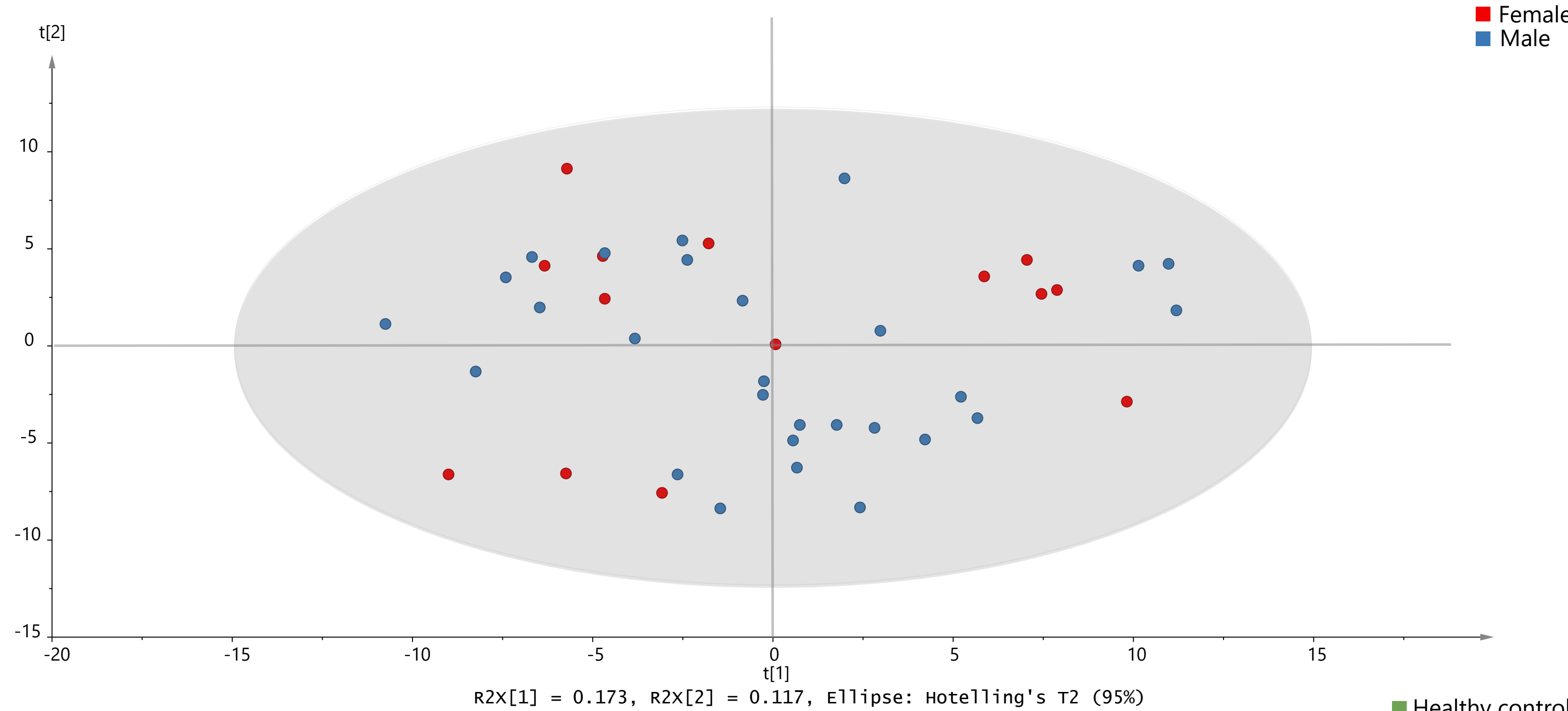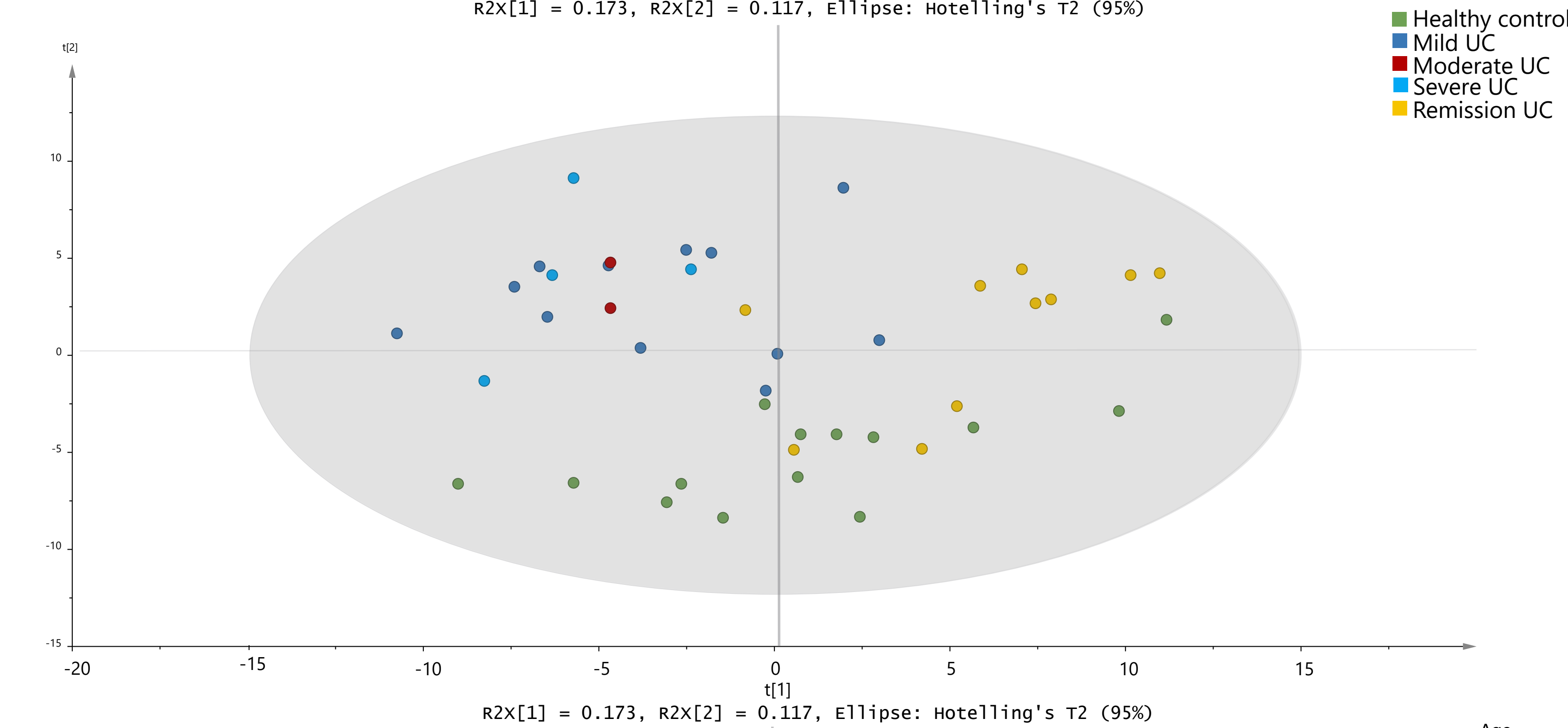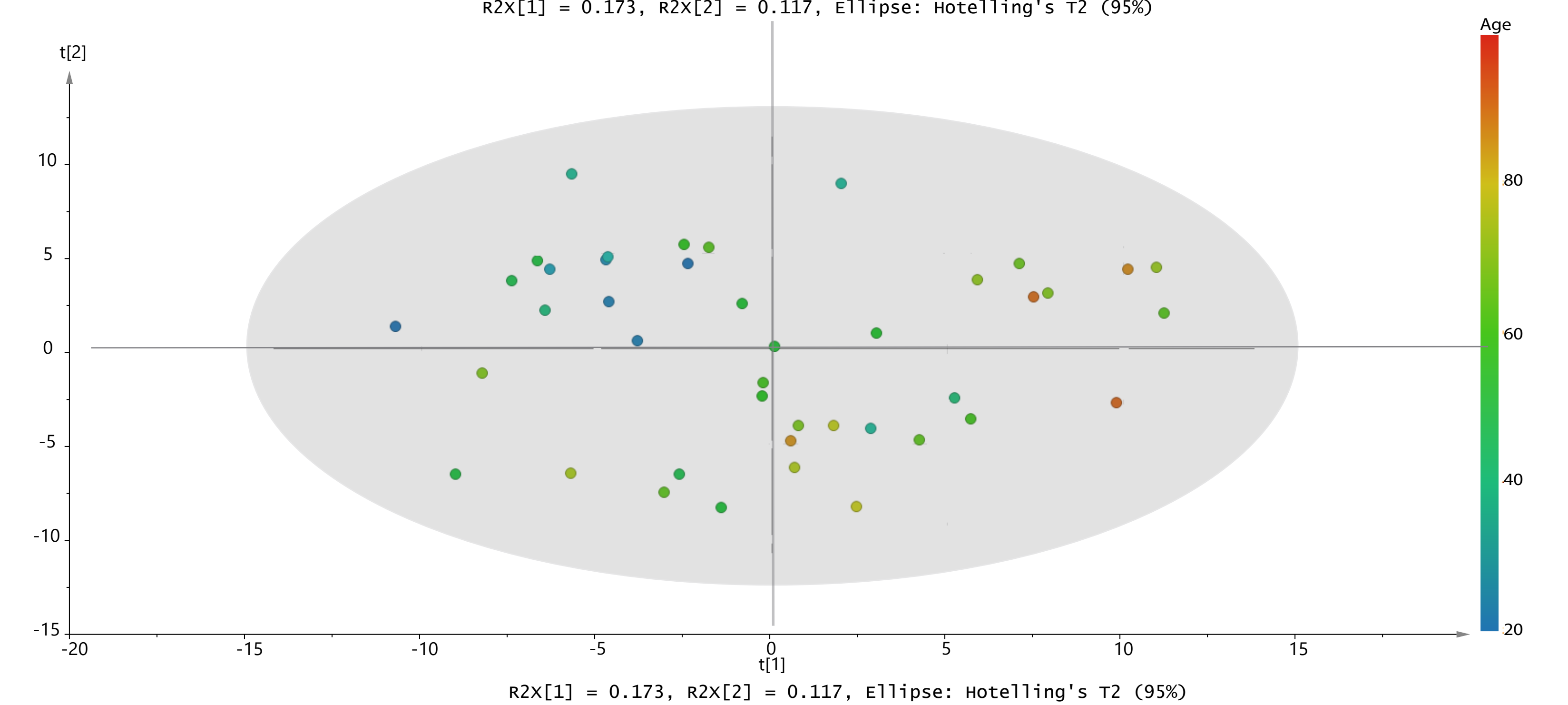

Supplement: Supplementary file 1 [file metabolites-09-00291-s001.zip › supplementary materials/Sup Figure 1.pdf]

R2Y(Cum), Q2(Cum)

● R2Y(Cum)  
■ Q2(Cum)

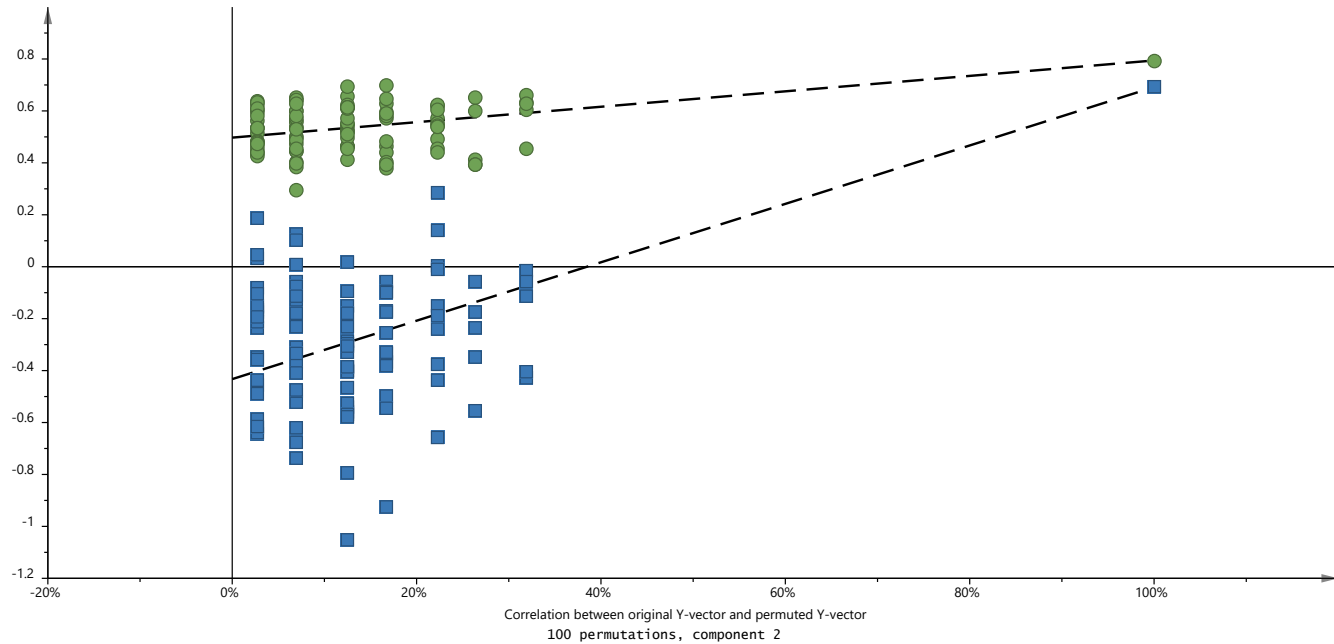

Supplement: Supplementary file 1 [file metabolites-09-00291-s001.zip › supplementary materials/Sup Figure 2.pdf]
